# Phenolic acids-rich fraction from *Ficus drupacea* leaves for the prevention and treatment of ethanol-induced gastric mucosal injury in rats

**DOI:** 10.1007/s10787-023-01158-4

**Published:** 2023-02-25

**Authors:** Sherien M. Bakry, Asmaa F. Aboul Naser, Sabri I. El Negoumy, Mona E. S. Kassem, Essam Abdel-Sattar, Meselhy R. Meselhy

**Affiliations:** 1 Phytochemistry and Plant Systematics Department, National Research Centre (NRC), 33 El Bohouth St. Dokki, Giza, 12622 Egypt; 2 Therapeutic Chemistry Department, National Research Centre (NRC), 33 El Bohouth St. Dokki, Giza, 12622 Egypt; 3 Pharmacognosy Department, Faculty of Pharmacy, Cairo University, Kasr El-Aini Street, Cairo, 11562 Egypt

**Keywords:** *Ficus drupacea*, Gastroprotection, Gastric mucosal injury, UPLC−PDA−ESI–MS/MS, Molecular networking, Phenolic acids

## Abstract

Bioactivity-guided fractionation of *F. drupacea* Thunb. extract revealed that the water fraction (FDWF) increased pH of the artificial gastric juice from 1.2 to 5.67 ± 0.015. The gastroprotective effect of FDWF against ulcer induced by ethanol was evaluated in rats. In ulcerogenic rats, increase in the gastric juice volume and ulcer lesions, and decrease in the gastric pH were evident. However, pretreatment with FDWF (100 mg/kg b.wt., p.o.) significantly inhibited lesion index, reduced gastric juice volume by 56.09% and increased gastric pH value. When given after ethanol, the same dose of FDWF led to significant healing of the gastric ulcer, with 75.60% reduction of gastric juice volume, and increase in pH value. In both prophylactic and therapeutic-treated groups, the level of superoxide dismutase and reduced glutathione in gastric homogenate were increased, while that of malondialdehyde was decreased. Also, the levels of succinate dehydrogenase and lactate dehydrogenase were increased, while that of acid phosphatase was decreased. In addition, the inflammatory markers; IL-10 and PGE2 were significantly increased. The histopathological results confirmed the above findings and indicated that the antiulcer effect of FDWF is mediated, at least in part, through antioxidant and anti-inflammatory mechanisms. Twenty-three compounds were tentatively identified in FDWF using UPLC−PDA−ESI–MS/MS and most of them were found to be phenolic acid derivatives. FDWF was standardized to contain 23.66 ± 2.62 mg/g and 8.86 ± 0.29 mg/g of quinic acid and chlorogenic acid, respectively. Accordingly, FDWF is a potential natural product that could increase the healing of gastric mucosal injury and prevents the development of ethanol-induced gastric mucosal injury in rats.

## Introduction

Gastric mucosal injury remains one of the most common diseases of GIT. The long-term use of nonsteroidal anti-inflammatory drugs, alcohol consumption, *H. pylori* infection, increased secretion of hydrochloric acid and pepsin, and stressful conditions are among the most aggressive factors for developing PU (Sowndhararajan and Kang 2013, Aboul Naser et al. 2020).

For gastric and duodenal ulcers, several drugs have been described, including anticholinergics, histamine H_2_-receptor antagonists, antacids, and proton pump inhibitors (Sowndhararajan and Kang 2013). Unfortunately, a series of adverse effects may result from prolonged use of these drugs (Sheen and Triadafilopoulos 2011). Recently, there is an increasing interest in developing plant-based agents for the effective management of PU (Patel et al. 2012).

Plants of genus *Ficus* are globally tropical ornamental trees, parts of which are used in folk medicine (Yessoufou et al. 2015), while the fruits of others, particularly *F. sycamorus* L. and *F. carica* L. are used as food in Egypt since ancient time (Manniche 1989).

Some *Ficus* spp*.* were studied for their antiulcer activity; the ethanolic extract of *F. carica* leaves exhibited antiulcer activity against chemical (HCl−Alcohol) and pylorus ligation-induced ulcers (Patil and Patil 2011). Moreover, the aqueous extract of *F. deltoidea* whole plant protect against ethanol-induced gastric ulcer (Fatimah et al. 2009) and the ethanolic extract of *F. religiosa* leaves decreases ulcer area and the volume of acidic secretion in a dose-dependent manner (Gregory et al. 2013). On the other hand, *F. drupacea* was used in folk medicine to treat sinusitis, malaria, and lung fluke. Moreover, the plant was used in the pharmaceutical industry as a source of *α*-glucosidase inhibitors (Phan et al. 2013). However, its protective effect against ethanol-induced gastric mucosal injury was not previously reported.

One of the animal models commonly used to investigate new antiulcer drugs is the ethanol-induced gastric ulcer (Arab et al. 2015; Sistani Karampour et al. 2019). Ethanol administration lowers the release of bicarbonate, gastric mucus, and nitric oxide while also causing stomach necrosis and subsequent infiltration of inflammatory cells. In addition, ethanol decreases stomach blood flow and causes oxidative stress by lowering glutathione production and elevating malondialdehyde formation (Aboul Naser et al. 2020).

This study was designed to evaluate the potential of *F. drupacea* extract/fractions as protective agent against ethanol-induced gastric mucosal injury in rat model. Ulcer indices, count, and pH value were recorded. The oxidative stress targets; reduced glutathione (GSH), superoxide dismutase (SOD), and malondialdehyde (MDA), inflammatory markers; interleukin-10 (IL-10) and prostaglandin E2 (PGE2), and relevant enzymes, such as succinate dehydrogenase (SDH), lactate dehydrogenase (LDH) and alkaline phosphatase (AP) were assessed. Histological features of the stomach were investigated. The metabolic profile and standardization of the bioactive fraction were performed using UPLC−PDA−ESI–MS/MS.

## Materials and methods

### Plant material

Sample from the leaves of *Ficus drupacea* Thunb., was collected from Al Zohriya botanical garden Cairo, Egypt in August 2020. The plant material was authenticated by Mrs. Trease Labib, Consultant of Plant Taxonomy at Ministry of Agriculture and the former director of El-Orman botanic garden. A voucher specimen (No. 2.10.2022. III) was deposited at the herbarium of the Department of Pharmacognosy, Faculty of Pharmacy, Cairo University. The plant material was dried in shade, powdered, and kept in closed container till use.

### Fractionation of the total extract of *F. drupacea* leaves

A total of 1.5 kg of powdered leaves was macerated with 70% ethanol (5 × 3L) for 5 days. The solution obtained was evaporated using Rotavapor® (Heidolph, Germany) to yield 80 g of dry extract. Part (40 g) of the extract was suspended in water (100 mL) and applied on the top of a Diaion HP20 (Sigma Aldrich, Supelco, USA) column (6 cm × 125 cm). Elution was started with water (5 L), 25% methanol/water (3 L), 50% methanol/water (3 L), 100% methanol (2.5 L), and dichloromethane (1 L). Fractions were evaporated under reduced pressure to yield 26, 4.8, 2.6, 1.0, and 0.5 g of dry residue, respectively.

### Determination of the neutralizing effect on artificial gastric acid

100 mg of each fraction or extract was weighed and dissolved in 250 ml of distilled water. The standards used in the proposed study were  the antacid-1 (sodium bicarbonate) and antacid-2  (a combination of aluminum hydroxide and magnesium hydroxide). The freshly prepared plant fractions (in 90 mL) or distilled water (90 mL) is added separately to the artificial gastric juice (100 mL) at pH 1.2. The pH values are determined to examine the neutralizing effects on artificial gastric juice.

For preparation of artificial gastric juice, two grams of NaCl and 3.2 mg of pepsin are dissolved in 500 mL distilled water. Hydrochloric acid (7.0 mL) and adequate water are added to make a 1000 mL solution. The pH of the solution is adjusted to 1.2 (Panda et al. 2017). A pH meter is connected continuously to monitor the changes of pH, and pH values were determined in triplicates.

### Antioxidant activity

The antioxidant activity of *F. drupacea* extract and FDWF was evaluated using DPPH (2,2-diphenyl-1-picryl-hydrazyl-hydrate) free radical scavenging assay (Shen et al. 2010). Samples were screened at 500 µg/ml using 0.1 mM DPPH dissolved in methanol. After an incubation for 30 min in the dark at room temperature, the absorbance was measured at 517 nm and a reference wavelength of 690 nm. Ascorbic acid was used as positive control at different concentration ranging from 11–2.7 µg/ml. The DPPH/methanol mixture was used as a negative control. The DPPH scavenging activity of samples was calculated according to the following equation:

$${\text{Percentage reduction }} = \, ({1} - ({\text{X}}/ \, \left( {{\text{av }}\left( {{\text{NC}}} \right)} \right) \, \times {1}00$$

where *x* indicates the absorbance of sample and av indicates the average absorbance of control and NC indicates the absorbance of negative control.

### In vivo evaluation of gastroprotective effect of FDWF

#### Chemicals

All chemicals used in this study were supplied by Sigma Co. (Saint Louis, USA). ELISA kits for interleukin-10 (IL-10) and prostaglandin E2 (PGE2) were provided by Cloud Clone Corp (Saint Louis, USA), and famotidine was a generous gift from Amon Co., Egypt.

#### Acute toxicity study

Forty male Wistar albino rats (100–120 g) were divided into four groups to assess acute toxicity at different plant concentrations (50, 100, and 200 mg/kg b.wt). For 15 days, the animals were monitored. There were no dead rats observed during the experiment, indicating that the extract was safe. As a result, a dose of 100 mg/kg b.wt was chosen for the in vivo study.

### Experimental design

Male Wistar albino rats (100–120 g) were obtained from the Animal House at Egypt's National Research Centre. All animals were kept in an air and temperature-controlled environment with access to water and standard pellets. Animal handling and their termination were carried out in accordance with the Medical Ethical Committee of the National Research Centre, Giza, Egypt. (Approval No. 20114).

Thirty-six male rats were divided into six groups (each of six rats). Rats in group 1 received distilled water (negative control group), while rats in group 2 received a daily dose of the FDWF (100 mg/kg b.wt, *p.o*) for one week. Rats in group 3 (the ulcerogenic rats) received a single dose of absolute ethanol (5 mL/kg b.wt, intragastric gavage) on a 24 h empty stomach, and sacrificed 1 h later (Mard et al. 2008; Eskander et al. 2021). Group 4 rats (the protective group) were given FDWF for 1 week prior to ulcer induction, while group 5 rats (the treatment group) were given a single oral dose of absolute ethanol on an empty stomach for 24 h and then after 1 h a daily dose of FDWF for 1 week. Group 6 rats were given absolute ethanol on an empty stomach for 24 h before receiving a daily dose of the reference antiulcer drug famotidine (20 mg/kg b.wt, *p.o*) for one week (Sen et al. 2013). After the last administration, all animals were fasted for 24 h, anesthetized by 1 ml of diethyl ether on piece of cotton (by inhalation) and sacrificed by decapitation.

#### Measurement of gastric content volume, pH, and number of gastric lesions

Just after anesthesia, the stomach contents of each rat were collected and centrifuged at 300 *g* for 15 min. The pH and volume of the supernatant were measured. After the stomach content was removed, the stomach was opened from the long curvature, washed with saline, expanded, and the number of lesions counted using a magnifying lens.

#### Tissue homogenate preparation

Longitudinal sections of each stomach weighing 0.5 g were homogenized in 5 mL of phosphate buffered saline (pH 7.4). The homogenate was centrifuged for 10 min at 300 *g* at 4 °C, and the supernatant was collected and kept at −20 °C for ongoing studies.

#### Determination of biochemical markers

The stomach oxidative stress targets were determined in all groups of animals using previously reported methods; GSH (Moron et al. 1979), MDA (Buege and Aust 1978), and SOD (Nishikimi et al. 1972). Also, level of enzymes such as SDH (mitochondria marker) (Shelton and Rice 1957), LDH (cytoplasm marker) (Babson and Babson 1973), and AP (lysosome marker) (Wattiaux and De Duve 1956) was measured. The ELISA technique was used to determine the inflammatory index in gastric tissue. PGE2 and IL-10 were measured using a solid phase competitive ELISA kit (Abcam, ab100765, Cambridge, USA), and a colorimetric assay was used to determine total protein in the stomach (Bradford 1976).

#### Histopathological examination

The stomach tissue slices were embedded in paraffin wax blocks after being fixed in 4% paraformaldehyde. Pathological changes were examined under a light microscope using 5-mm thick sections stained with hematoxylin and eosin (H&E) (Hirsch et al. 1997).

#### Statistical analysis

The data were presented as the mean ± standard deviation (SD) of six rats in each group. For statistical analysis, one-way analysis of variance (ANOVA), Costat Software Computer Program, with post hoc test at least significance difference (LSD) between groups at *p* < 0.05, were used.

$$\% {\text{ Of change }} = \frac{{{\text{Control mean }} - {\text{ Treated mean}}}}{{\text{Control mean}}}$$

$$\% {\text{ Of improvement }} = \left[ {\left( {{\text{Mean of ulcer group }} - {\text{ mean of }}\left( {\text{treated or prophylactic group}} \right)} \right) \, /{\text{ mean of healthy group}}} \right]{\text{ x 1}}00.$$

#### UPLC−PDA−ESI–MS/MS analysis of *F. drupacea* active fraction (FDWF)

The analysis was performed on a MaXis-4G instrument. (Bruker Daltonics, Bremen, Germany) linked to an Ultimate 3000 HPLC (Thermo Fisher Scientific). UPLC condition were performed as described before by (Hegazi et al. 2020). The parameters used for the tandem MS follows (Garg et al. 2015). Sodium formate was used as a calibrant, and the acquired data was calibrated using a Bruker-developed script.

### Data analysis

Compass Data Analysis 4.4 (Bruker Daltonics®, Germany) was used for raw data visualization, while Metaboscape 3.0 (Bruker Daltonics®) was used for molecular feature selection, raw data treatment, and preprocessing. The T-ReX 3D (Time aligned Region Complete eXtraction) algorithm was used to detect and combine isotopes, adducts, and fragments innate to the same compound into a single feature. After that, a bucket table was created that contained all of the detected features as well as their retention time, measured m/z, molecular weight, and detected ions (Olmo‐García et al., 2019). The catalogued ions table was created with a negative ionization mode intensity threshold of 10e^3^, a retention time range of 1 to 40 min, and a restricted mass range m/z of 120–1800 Da.

### Molecular networking

The MS^2^ data were independently uploaded as an mgf file to the publicly accessible Global Natural Product Social molecular networking (GNPS) platform (http://gnps.ucsd.edu, accessed on 31 July 2022) running the online feature-based molecular networking workflow (Wang et al. 2016).

The data were analyzed with a parent mass tolerance of 0.05 Da and an MS/MS fragment ion tolerance of 0.05 Da to generate consent spectra. A network with a cosine score of 0.7 and more than 6 matched peaks between two consensus mass spectra linked by an edge was then built. The network was compared to GNPS spectral libraries (NIST13, MassBank, and Respect). The molecular network and parameters can be found at the following link: https://gnps.ucsd.edu/ProteoSAFe/status.jsp?task=8a25fad020c6409c8a577cbe9fdecb59.

Consequently, the molecular network that was built was enhanced with a MolNetEnhancer to provide a more comprehensive chemical overall view of metabolomics data. (Ernst et al. 2019). Cytoscape was used to visualize and analyze the output molecular network (3.9.1) (Shannon et al. 2003).

### Total phenolic content (TPC)

Phenolic compounds (TPC) of *F. drupacea* extract and FDWF were determined by Folin−Ciocalteu reagent according to the method of (Antolovich et al. 2002) with minor modifications. In brief, 20 μL of samples were mixed with 100 μl of 1:10 Folin−Ciocalteu reagent, then Na_2_CO_3_ (80 L, 7.5%) was added. After incubating at room temperature for 2 h in the dark, the absorbance at 690 nm was measured in a microplate. The standard reference was gallic acid. TPC (total phenolic content) was measured in mg of gallic acid equivalents per g of dried extract (mg GAE g^−1^) (gallic acid equivalent).

### Quantification of quinic acid and chlorogenic acid

The sample was analyzed with liquid chromatography−electrospray ionization−tandem mass spectrometry (LC–ESI–MS/MS) using an Exion LC AC system for separation and a SCIEX Triple Quad 5500 + MS/MS system with ESI for detection. For the separation, a ZORBAX Eclipse Plus C18 Column (4.6100 mm, 1.8 m) was used. The mobile phase was 0.1% formic acid in water (solvent A) and acetonitrile (solvent B) with the following gradient: 2% B from 0–1 min, 2–60% B from 1–8 min, 60% B from 8–12 min, and 2% B from 12.01–15 min. The flow rate was 0.8 mL/min and the injection volume was 3 µL. The selected polyphenols were analyzed using the negative ionization mode using Multiple Reaction Monitoring (MRM). The curtain gas was 25 psi, the ion spray voltage was 4500, the source temperature was 400 °C, the ion source gases 1 and 2 were 55 psi with a declustering potential of 50, the collision energy was 25 eV and the collision energy spread was 10 eV.

## Results

### Bioactivity-guided fractionation of total extract of *F. drupacea* leaves

Fractionation of the total extract using Diaion^@^ HP20 column afforded 5 fractions. Their acid neutralizing effects revealed that the water fraction (FDWF) has the most neutralizing effect (1.96 ± 0.015), followed by the 75% methanol fraction (1.81 ± 0.01) (Table 1). The antacids-1 and 2 showed neutralizing effects of 1.81 ± 0.01 and 1.66 ± 0.01, respectively). Therefore, FDWF was selected for the in vivo study.

**Table 1:** In vitro neutralizing effects of different fractions of *F. drupacea*

Sample	pH	Neutralizing effect
Water fraction (FDWF)	5.67 ± 0.015	1.96 ± 0.015
25% Methanol fraction	5.21 ± 0.021	1.70 ± 0.01
50% Methanol fraction	3.33 ± 0.01	1.64 ± 0.01
75% Methanol fraction	5.00 ± 0.025	1.81 ± 0.01
100% Methanol fraction	4.66 ± 0.02	1.72 ± 0.02
Antacid-1	7.80 ± 0.05	1.81 ± 0.015
Antacid-2	7.40 ± 0.05	1.66 ± 0.015
Water	6.80 ± 0.05	1.55 ± 0.042

^*^All results are expressed as mean ± SD (*n* = 3)

### Antioxidant activity

FDWF showed higher DPPH radical scavenging activity [IC_50_ value of 231 ± 0.074 µg/ml] compared with total extract [IC_50_ value of 516 ± 0.086 ug/ml].

### In vivo study of FDWF on ethanol-induced gastric mucosal injury in rats

#### Effect on ulcer index

The volume of gastric juice in normal rats was found to be 0.17 ± 0.05 mL However, a drastic increase in the gastric juice volume to 2.05 ± 0.42 mL (+ 1105.88%) was noted in the ulcerogenic group rats (Table 2). When compared with that of ulcerogenic rats, a marked decline in gastric volume by 56.09 and 75.60%, respectively, was evident after administration of FDWF before (prophylactic group, G-4) or after ethanol (therapeutic-treated group, G-5) to rats, with respective amelioration percentages of 676 and 911%. In comparison to the control group, the pH value of the gastric juice in ulcerogenic rats was reduced by 27.47% (Table 2). When compared with the ulcerogenic group, both FDWF-prophylactic and therapeutic-treated rats, as well as the famotidine-treated group, showed insignificant increase of 12.77, 24.45, and 19.04%, respectively. As a result, the healing improvement values were 7.84, 17.7, and 13.81%, respectively.

**Table 2:** Effect of FDWF on gastric ulcer index

	Groups	Gastric volume (mL) (% change)	pH (% change)	Ulcer lesion count (% change)
G-1	Control	0.17^c^ ± 0.05	6.37 ± 0.25^a^	–
G-2	Normal + FDWF	0.19^c^ ± 0.05 (+ 11.76)	6.25 ± 0.28^a^ (−1.88)	–
G-3	Ulcerogenic rats	2.05^a^ ± 0.42 (+ 1105.88)	4.62 ± 0.75^c^ (−27.47)	7.25^a^ ± 0.95
G-4	Prophylactic ulcerogenic rats + FDWF	0.90^b^ ± 0.17 [−56.09]	5.12^bc^ ± 0.25 [+ 12.77]	4.00^b^ ± 0.81 [−44.42]
G-5	Ulcerogenic rats + FDWF	0.50^c^ ± 0.08 [−75.60]	5.75 ± 0.29^ab^ [+ 24.45]	2.50^c^ ± 0.57 (−65.51)
G-6	Ulcerogenic rats + Famotidine	0.41^c^ ± 0.07 [−80.00]	5.50 ± 0.40^b^ [+ 19.04]	2.00^c^ ± 0.81 (−72.41)

The data are presented as the mean ± SD of six rats in each group

Groups with the same letters are nonsignificantly different, whereas groups with different letters are significantly different (*p* < 0.05)

The values between the brackets represent percentage changes compared to the control group

The percentage changes versus the ulcer group are shown in parentheses

**Fig. 1:**
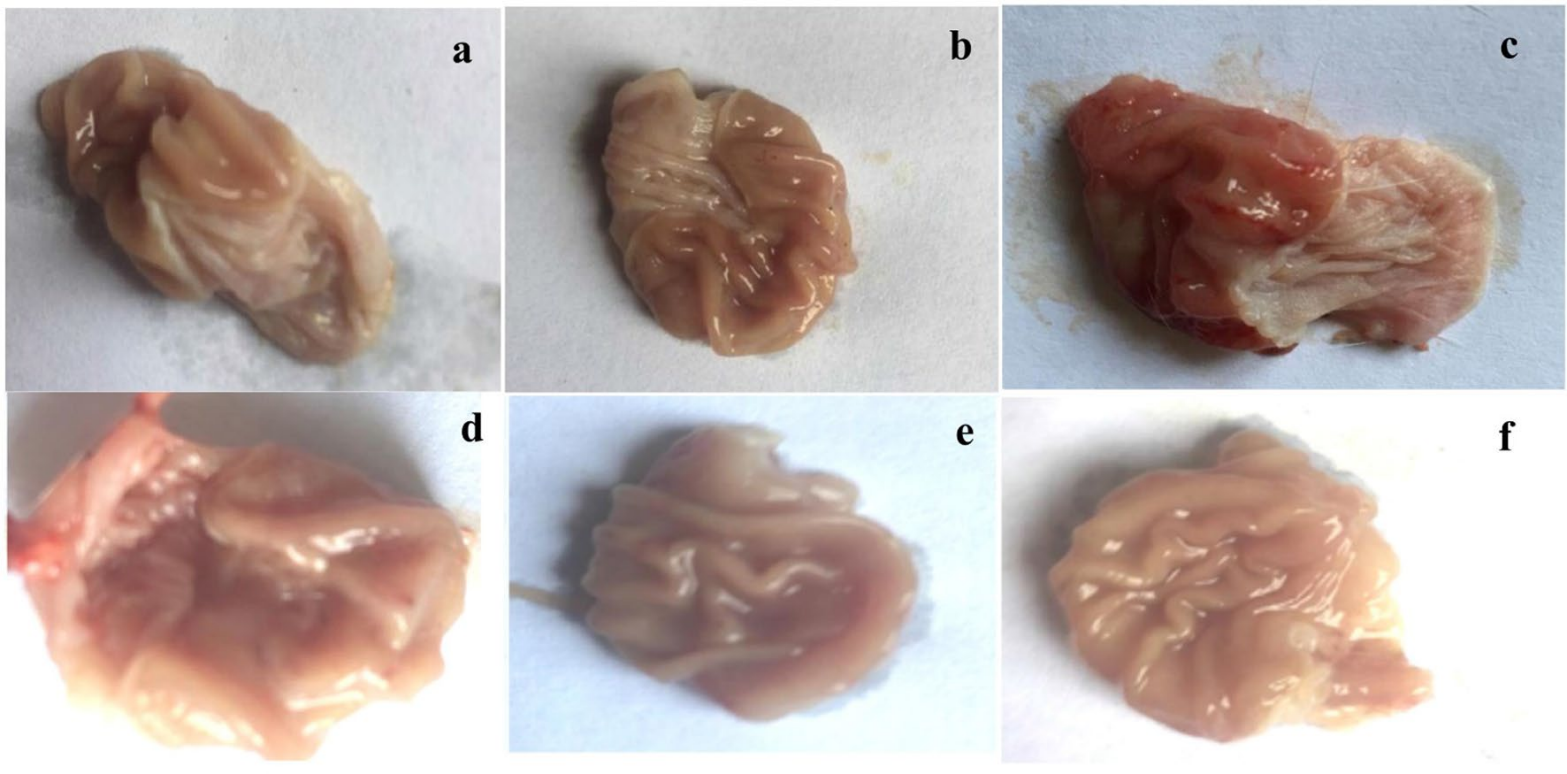
Gross appearance of rat gastric mucosa. **A** Gastric mucosa of normal control group, **B** control FDWF group, **C** ulcerogenic group, **D** prophylactic group, **E** treated group with FDWF, **F** famotidine-treated group

Furthermore, the ulcerogenic rats had seven lesions on average per stomach. When compared with the ulcerogenic rats, those in the FDWF-prophylactic and therapeutic-treated groups, as well as the famotidine-treated group showed significant decrease of 44.42, 65.51, and 72.41%, respectively (Fig. 1).

#### Effect on cell organelles marker enzymes

The levels of SDH, LDH, and AP in mucosal tissues of rats in the control group before (G-1) or after administration of FDWF (G-2) were insignificantly different (Table 3).

**Table 3:** Effect of FDWF on cell organelles marker enzymes

	Groups	SDH (mmol/mg protein)	LDH (mmol/mg protein)	AP (mmol/mg protein)
G-1	Control	8.01^a^ ± 0.59	8.11^a^ ± 0.84	18.16^c^ ± 2.09
G-2	Normal + FDWF	6.68^a^ ± 0.29 (−16.60)	7.78^a^ ± 0.26 (−2.95)	17.77^c^ ± 0.26 (−2.14)
G-3	Ulcerogenic rats	2.77^e^ ± 0.74 (−65.41)	3.15^c^ ± 0.23 (−61.15)	43.76^a^ ± 9.02 (+ 140.96)
G-4	Prophylactic ulcerogenic rats + FDWF	4.12^d^ ± 0.75 [+ 48.73]	5.35^b^ ± 0.72 [+ 69.84]	32.27^b^ ± 1.98 [−26.25]
G-5	Ulcerogenic rats + FDWF	5.17^c^ ± 0.39 [+ 86.64]	5.53^b^ ± 0.49 [+ 75.55]	24.32^b^ ± 1.26 [−44.42]
G-6	Ulcerogenic rats + famotidine	4.39cd ± 0.43 [+ 58.48]	5.30^b^ ± 0.50 [+ 68.25]	26.02^b^ ± 2.83 [−40.53]

The data are presented as the mean ± SD of six rats in each group

Groups with the same letters are nonsignificantly different, whereas groups with different letters are significantly different (*p* < 0.05)

The values between the brackets represent percentage changes compared to the control group

The percentage changes versus the ulcer group are shown in parentheses

However, a significant decrease in the levels of both SDH and LDH (65.41 and 61.15%, respectively), and a significant increase in AP level (140.96%) were observed in the ulcerogenic rats (G-3), when compared with the control group. On the other hand, a significant improvement in the level of SDH was observed after administration of FDWF [for protection (G-4) or treatment (G-5)], or famotidine (G-6); improvement percentages of 16.85, 29.96, and 20.22%, respectively. Similarly, LDH level was improved in the FDWF or famotidine groups (with amelioration values of 27.12, 29.34, and 26.51%, respectively), when compared with the ulcerogenic rats. However, a reduction in AP level in ulcerogenic rats (G-3) was observed in both the prophylactic and therapeutic-treated groups, with improving values of 63.27, 107.04, and 97.68%, respectively (Table 3).

#### Effect on oxidative stress markers

There were no significant changes in oxidative stress markers or total protein content after administering FDWF to normal rats (Table 4). Rats with stomach ulcers had a 72.09% reduction in GSH level when compared to the control group, whereas those with ulcers who were protected or treated with FDWF or famotidine had a significant increase of 91.18, 123.18, and 142.70%, respectively, when compared to the ulcerogenic group. This corresponded to a level of improvement of 25.44, 34.37, and 39.82%, respectively. Similarly, the ulcerogenic rats had a 70.49% decrease in SOD levels when compared to the healthy group. The administration of the FDWF (for protection or treatment) or famotidine to ulcerogenic rats resulted in an increase of SOD level (78.02, 127.82, and 123.64%, respectively), when compared with the ulcerogenic group. In contrast, ulcerogenic rats had a significant increase in MDA level by 477.98% when compared to normal rats, whereas those exposed to both protection and treatments had a significant decrease in MDA level by 27.34, 53.19, and 56.64%, respectively, when compared to ulcerogenic rats. As a result, MDA recorded improvement values of 158.02, 307.45, and 324.88%, respectively. In comparison to the control group, ethanol caused a 219.60% increase in total protein levels. When compared with the ulcerogenic rats, it showed a reduction level of 25.19, 60.55, and 49.10% after protection and treatments with FDWF or famotidine, respectively. As a result, the improvement values were 80.48, 139.49, and 156.91%, respectively.

**Table 4:** Effect of FDWF on oxidative stress markers

	Groups	GSH (μg/g tissue)	MDA (mmol/mg protein)	SOD (U/g tissue)	Tissue protein (μg/g)
G-1	Control	34.15^a^ ± 2.20	8.72^d^ ± 0.76	85.13^a^ ± 3.44	30.75^e^ ± 1.26
G-2	Normal + FDWF	31.33^a^ ± 1.71 (−8.25)	8.10^d^ ± 0.65 (−7.11)	81.67^a^ ± 4.11 (−4.06)	31.50^e^ ± 1.91 (+ 2.44)
G-3	Ulcerogenic rats	9.53^d^ ± 1.35 (−72.09)	50.40^a^ ± 7.54 (+ 477.98)	25.12^d^ ± 1.49 (−70.49)	98.25^a^ ± 2.36 (+ 219.60)
G-4	Prophylactic ulcerogenic rats + FDWF	18.22^d^ ± 1.18 [+ 91.18]	36.62^b^ ± 5.94 [−27.34]	44.72^c^ ± 5.86 [+ 78.02]	73.50^b^ ± 4.72 [−25.19]
G-5	Ulcerogenic rats + FDWF	21.27^c^ ± 1.77 [+ 123.18]	23.59^c^ ± 1.09 [−53.19]	57.23^b^ ± 1.39 [+ 127.82]	38.75^d^ ± 4.85 [−60.55]
G-6	Ulcerogenic rats + Famotidine	23.13 ^c^ ± 2.09 [+ 142.07]	22.07^c^ ± 1.93 [−56.64]	56.18^b^ ± 2.51 [+ 123.64]	50.00^c^ ± 3.26 [−49.10]

The data are presented as the mean ± SD of six rats in each group

Groups with the same letters are not significantly different, whereas groups with different letters are significantly different (*p* < 0.05)

The values between the brackets represent percentage changes compared to the control group

The percentage changes versus the ulcer group are shown in parentheses

#### Effect on inflammatory markers

The administration of FDWF to normal rats resulted in insignificant changes in the level of IL-10 and PGE2. Rats given ethanol had a 33.12% decrease in IL-10 level when compared with the control group, whereas those with ulcers and exposed to either protection or treatments with FDWF or famotidine had an increase of 12.33, 23.40, and 31.53%, respectively, when compared with the ulcerogenic group. These observations revealed a level of improvement of 8.42, 15.65, and 21.09%, respectively. Similarly, the ulcerogenic rats showed a 41.63% reduction in PGE2 level when compared to the control group. PGE2 level was increased by 45.22, 54.09, and 62.42% after administration of FDWF for either protection or treatment, as compared to the ulcerogenic group; with improvement levels of 26.39, 31.57, and 36.43% in the prophylactic, therapeutic treated, and reference groups, respectively. (Table 5).

**Table 5:** Effect on inflammatory markers

	Groups	IL-10 (pg/mg)	PGE2 (pg/mg)
G-1	Control	124.75^a^ ± 4.47	174.05^a^ ± 7.12
G-2	Normal + FDWF	127.67^a^ ± 5.83 (+ 2.34)	178.17^a^ ± 6.05 (+ 2.36)
G-3	Ulcerogenic rats	83.43^d^ ± 5.50 (−33.12)	101.58^d^ ± 11.09 (−41.63)
G-4	Prophylactic ulcerogenic rats + FDWF	93.72^c^ ± 5.50 [+ 12.33]	147.52^c^ ± 8.70 [+ 45.22]
G-4	Ulcerogenic rats + FDWF	102.96^b^ ± 5.51 [+ 23.40]	156.53^bc^ ± 7.19 [+ 54.09]
G-5	Ulcerogenic rats + famotidine	109.74^b^ ± 6.49 [+ 31.53]	164.09^ab^ ± 4.75 [+ 62.42]

The data are presented as the mean ± SD of six rats in each group

Groups with the same letters are nonsignificantly different, whereas groups with different letters are significantly different (*p* < 0.05)

The values between the brackets represent percentage changes compared to the control group

The percentage changes versus the ulcer group are shown in parentheses

### Histopathological observations

Both control and normal rats given FDWF showed intact gastric mucosa, normally distributed gastric glands lined by mucus-secreting cells with rounded nuclei, and normal lamina propria (see Fig. 1A, 1B, as well as 2A, B).

**Fig. 2:**
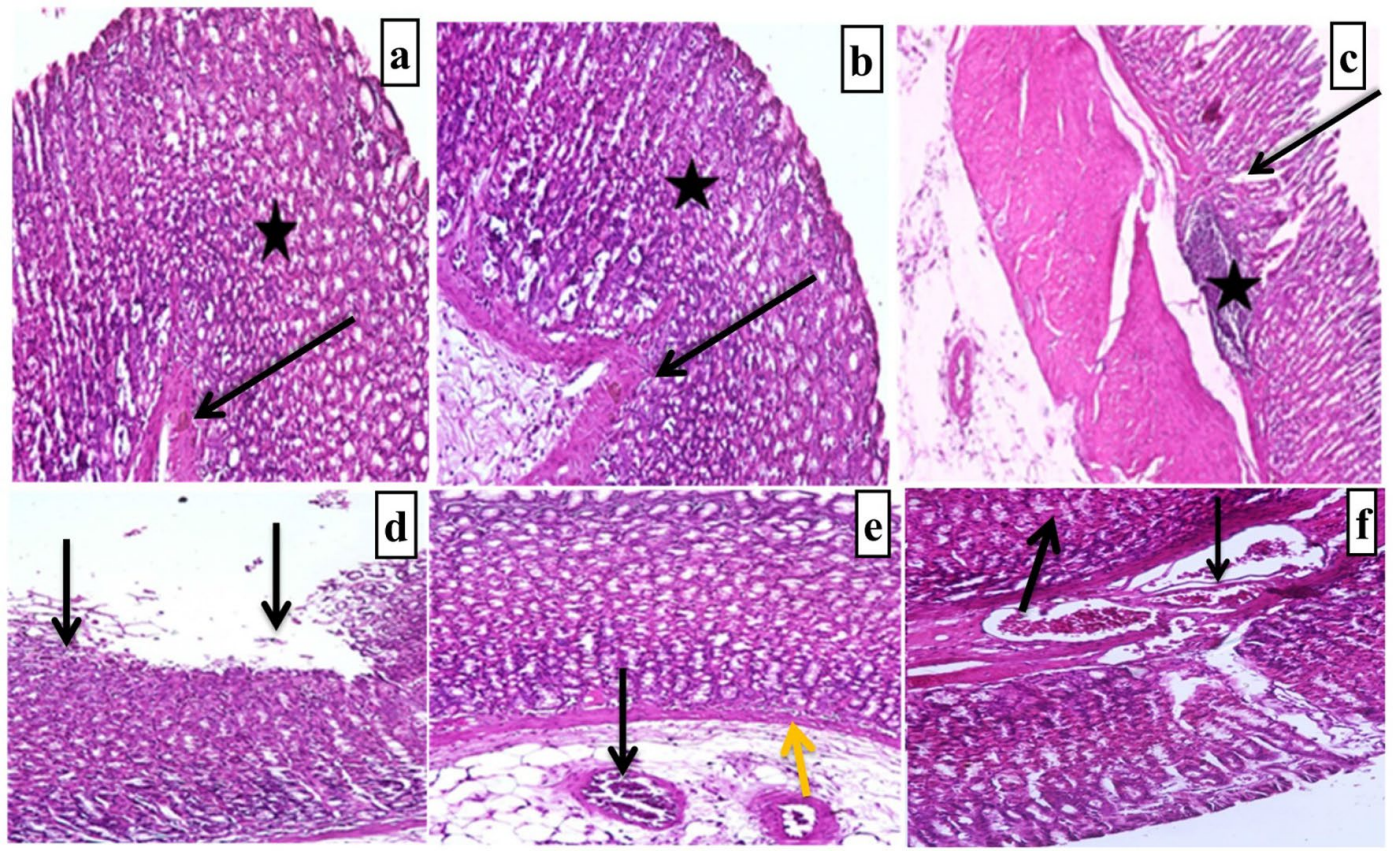
Photomicrographs of the gastric mucosa of control and FDWF-treated rats **A**, **B** showing normal stomach layers; note the normal mucosal (*), and submucosal epithelium and intact basement membrane (arrows), **C** ulcerogenic group showing ulcers; note the ulcer formation represented by deep mucosal layer (*) and destruction reaching the submucosal layer (black arrow), **D** prophylactic group with FDWF showing gastric erosion; note the superficial mucosal layer destruction with intact basement membrane (black arrows). **E** Treated group with FDWF showing normal stomach layers with absence of ulcer and inflammatory cells (yellow arrow), and some blood congestion (black arrow), (F) The famotidine-treated group had a healed ulcer with a moderately developed mucosal lining and less thickening than the control group (black arrows). All specimens were stained with haematoxylin and eosin (H& E) and viewed at a magnification of × 400

Hemorrhage and necrosis were observed in the gastric mucosa of ulcerogenic rats, resulting in severe submucosal odema and epithelial cell injury. At the ulcer's base, polymorphous lymphocytes were observed, and hyperplastic gastric glands surrounded the ulcer. The lamina propria, as well as fibrotic tissues, contained lymphocytes and polymorphonuclear leucocytes (see Figs. 1C, 2C)

Gastric erosion as well as superficial mucosal layer destruction in the presence of an intact basement membrane were observed in the FDWF-prophylactic group (Figs. 1D, 2D).

Ulcerogenic rats treated with FDWF showed completely healing of ulcer with well-developed and normal thickened mucosal membrane (Figs. 1F, 2F).

### UPLC−MS metabolic profile of FDWF

**Fig. 3:**
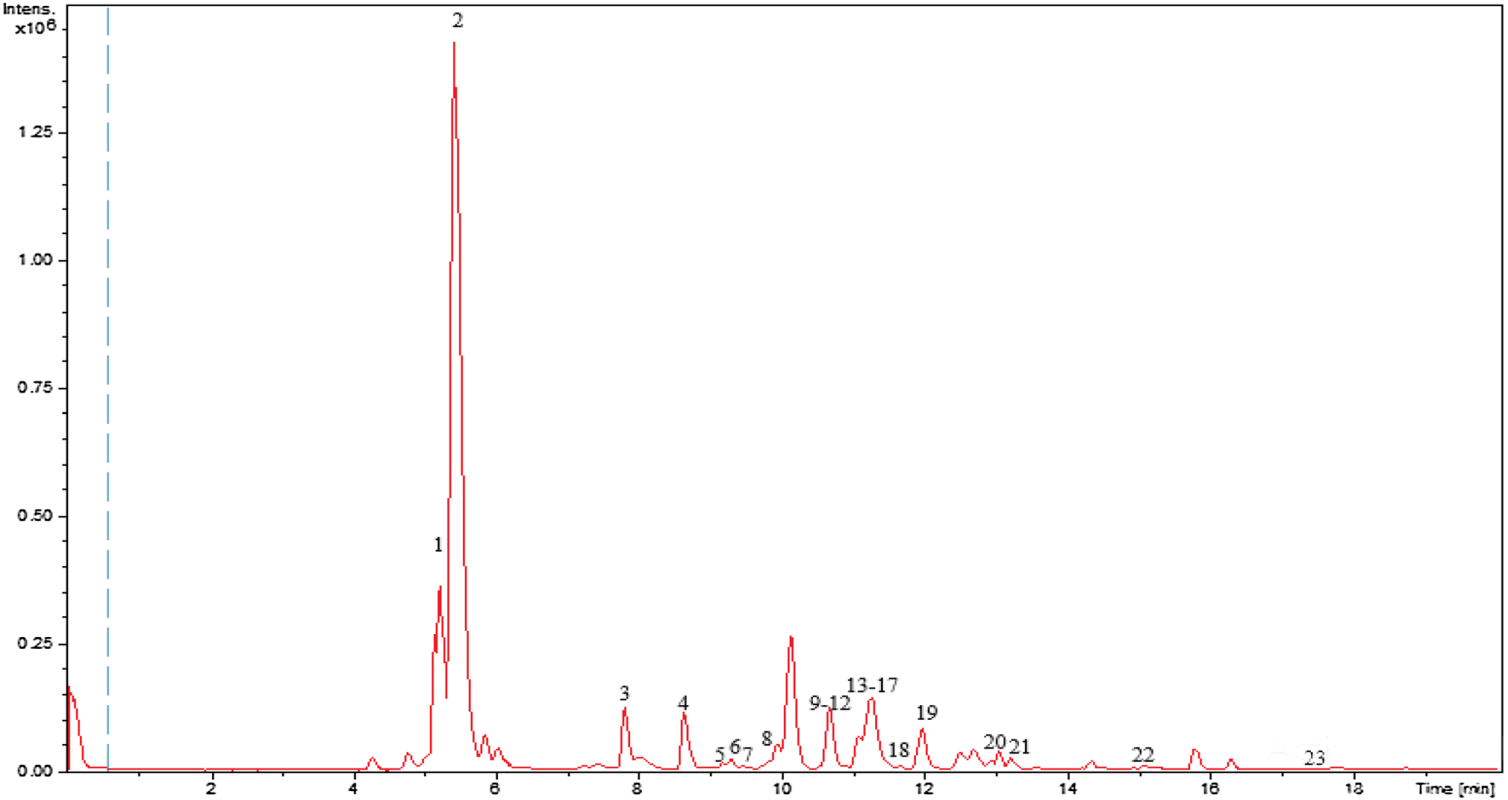
LC–MS base peak chromatogram of *Ficus drupaacea* water fraction

**Fig. 4:**
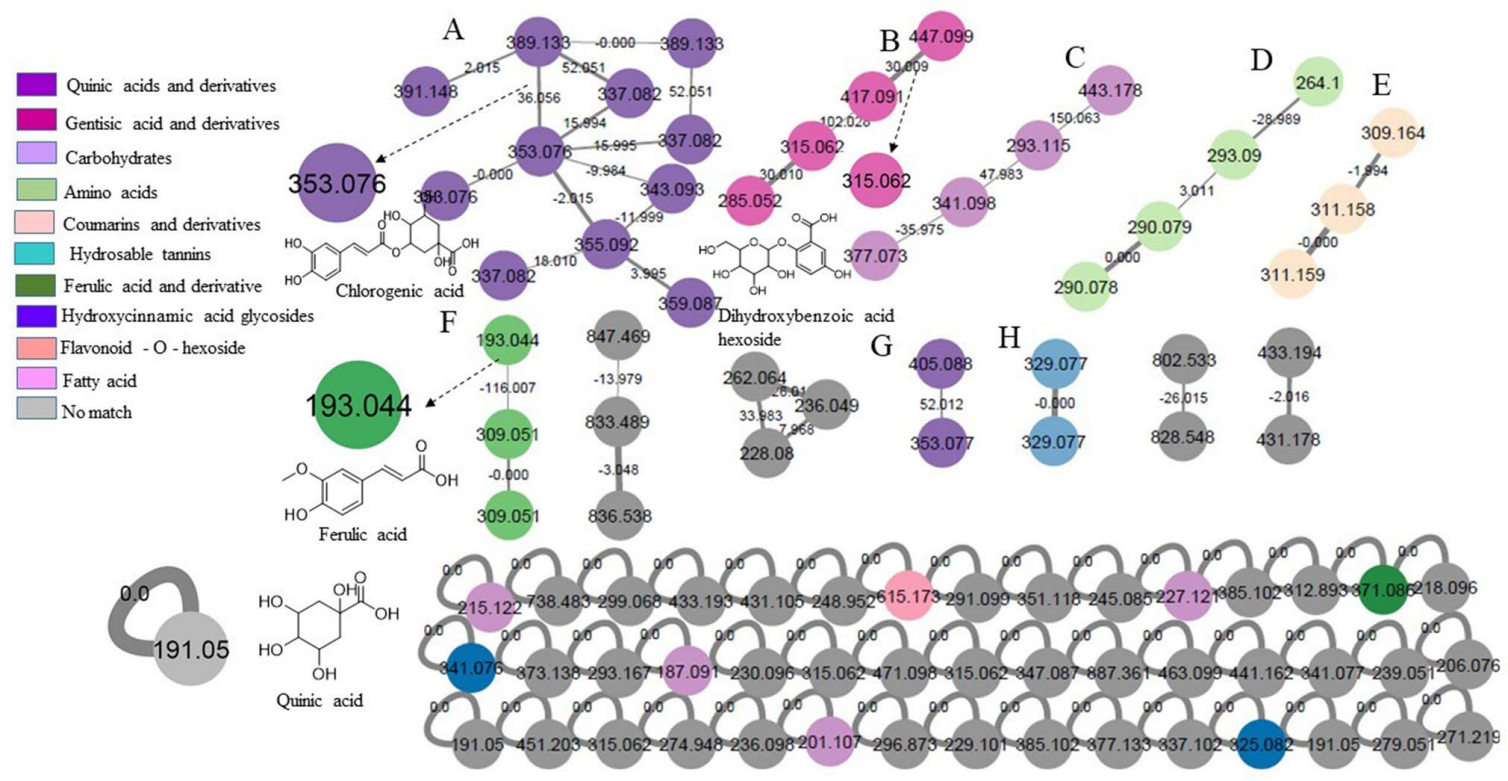
Full molecular networking (created using negative MS/MS data)

A total of 23 metabolites were tentatively identified in FDWF based on their retention time (rt), molecular formula, and fragmentation pattern (Fig. 3; Table 6). Phenolic acid derivatives were dominating FDWF with quinic acid as the major metabolite (Fig. 4).

**Table 6:** Tentatively identified metabolites in FDWF

No.	Rt	Metabolite	[M–H]^−^	MS^n^	Molecular formula	Error (ppm)	Refs.
1	5.2	Sucrose	341.0882	179.0551, 161.0457, 143.0352, 119.0349, 101.0247, 89.0245, 71.0140	C_12_H_22_O_11_	−0.1	Jin et al. (2018), GNPS
2	5.4	Quinic acid#	191.0561	127.0400, 85.0296	C_7_H_12_O_6_	−0.2	Chansriniyom et al. (2021), GNPS
3	7.9	Galloyl-rhamnopyranose	315.0719	169.0143, 125.0243	C_13_H_16_O_9_	−0.2	Abou-Zaid and Nozzolillo (1999)
4	8.7	Galloyl-rhamnopyranose	315.0723	169.0143, 125.0244	C_13_H_16_O_9_	−0.4	Abou-Zaid and Nozzolillo (1999)
5	9.2	Vanillic acid hexoside#	329.0893	167.0351, 151.0111, 123.0443	C_14_H_18_O_9_	0.2	Ammar et al. (2015)
6	9.3	Dihydroxybenzoic acid hexoside #	315.0716	153.0116, 108.0221	C_13_H_15_O_9_	0.2	Ammar et al. (2015)
7	9.5	Dihydroxybenzoic acid pentoside hexoside #	447.1141	315.0712, 152.0113	C_18_H_24_O_13_	0.9	Ammar et al. (2015)
8	9.6	Syringic acid hexoside#	359.0982	197.0450,191.0560, 184.0618,182.0212, 173.0455,153.0557, 138.0320	C_15_H_20_O_10_	0.2	Ammar et al. (2015)
9	10.6	Dihydroxybenzoic acid pentoside #	285.0612	153.0112, 108.0218	C_12_H_14_O_8_	−0.3	Farag et al. (2014)
10	10.7	Dihydroxybenzoic acid dipentoside #	417.1038	285.0619, 241.0710, 152.0115, 109.0289	C_17_H_22_O_12_	−0.3	Ammar et al. (2015)
11	10.8	Dihydrocaffeic acid hexoside #	343.1036	181.0505,137.0609, 135.0452	C_15_H_20_O_9_	−0.4	Ammar et al. (2015)
12	10.9	Caffeic acid hexoside	341.0874	179.0351, 135.0453	C_15_H_18_O_9_	1.3	Schütz et al. (2005)
13	11.1	Dihydroxybenzoic acid#	153.0191	109.0296, 108.0215, 81.0344	C_7_H_6_O_4_	1.6	Farag et al. (2014)
14	11.2	Coumaroyl Hexoside#	325.0929	164.0441, 119.0501	C_15_H_18_O_8_	−0.1	Ammar et al. (2015)
15	11.3	Coumaroylquinic acid#	337.0927	191.0562, 163.0400, 119.0500	C_16_H_18_O_8_	0.7	Ammar et al. (2015)
16	11.4	Chlorogenic acid (Caffeoylquinic acid) #	353.0877	191.0559	C_16_H_18_O_9_	0.3	Ammar et al. (2015), GNPS
17	11.5	Ferulic acid hexoside #	355.1031	193.0508,191.0560, 178.0266, 134.0374	C_16_H_20_O_9_	1	Ammar et al. (2015)
18	11.6	Feruloylquinic acid #	367.1032	193.0509, 191.0555, 134.0373	C_17_H_20_O_9_	0.8	Fang et al. (2002)
19	12	Chlorogenic acid (Caffeoylquinic acid) #	353.0881	191.0563	C_16_H_18_O_9_	−0.8	Ammar et al. (2015), GNPS
20	13.3	Coumaroylquinic acid#	337.0927	191.0562, 163.0400, 119.0500	C_16_H_18_O_8_	0.4	Ammar et al. (2015), GNPS
21	13.5	Sinapic acid hexoside#	385.1109	267.0733, 249.0613	C_17_H_22_O_10_	−0.2	Ammar et al. (2015)
22	15.1	Ferulic acid malate#	309.0613	193.0508,178.0275,149.0599, 134.0372; 115.0030	C_14_H_14_O_8_	1	Ammar et al. (2015)
23	17.6	Ferulic acid#	193.0505	161.0245, 134.0374	C_10_H_10_O_4_	−0.0	Ammar et al. (2015)

^#^Identified from the same genus

Quinic acid derivatives grouped in clusters **A** and **G** were identified as coumaroylquinic acid (**15, 20**), chlorogenic acid (**16, 19**) and feruloylquinic acid (**18**); their MS2 spectra shared the same fragment ion at *m/z* 191 amu for quinic acid moiety. Dihydroxybenzoic acid derivatives grouped in cluster **B** were annotated as dihydroxybenzoic acid hexoside (**6**), dihydroxybenzoic acid pentoside hexoside (**7**), dihydroxybenzoic acid pentoside (**9**) and dihydroxybenzoic acid dipentoside (**10**). Derivatives of ferulic acid grouped in cluster **F** were identified as ferulic acid malate (**22**) and ferulic acid (**23**). Cluster **D** represents amino acids, **E** represents coumarins and derivatives, and **H** represents hydrolysable tannins. Hydroxycinnamic acid glycosides are selflooped annotated as caffeic acid hexoside (**12**) and coumaroyl hexoside (**14**).

### Total phenolic content (*TPC*)

The contents of phenolics in the total extract of *F. drupacea* extract and FDWF were found to be 42 ± 0.55 and 52.3 ± 1.12, respectively.

### Quantification of quinic acid and chlorogenic acid using LCMS

**Fig. 5:**
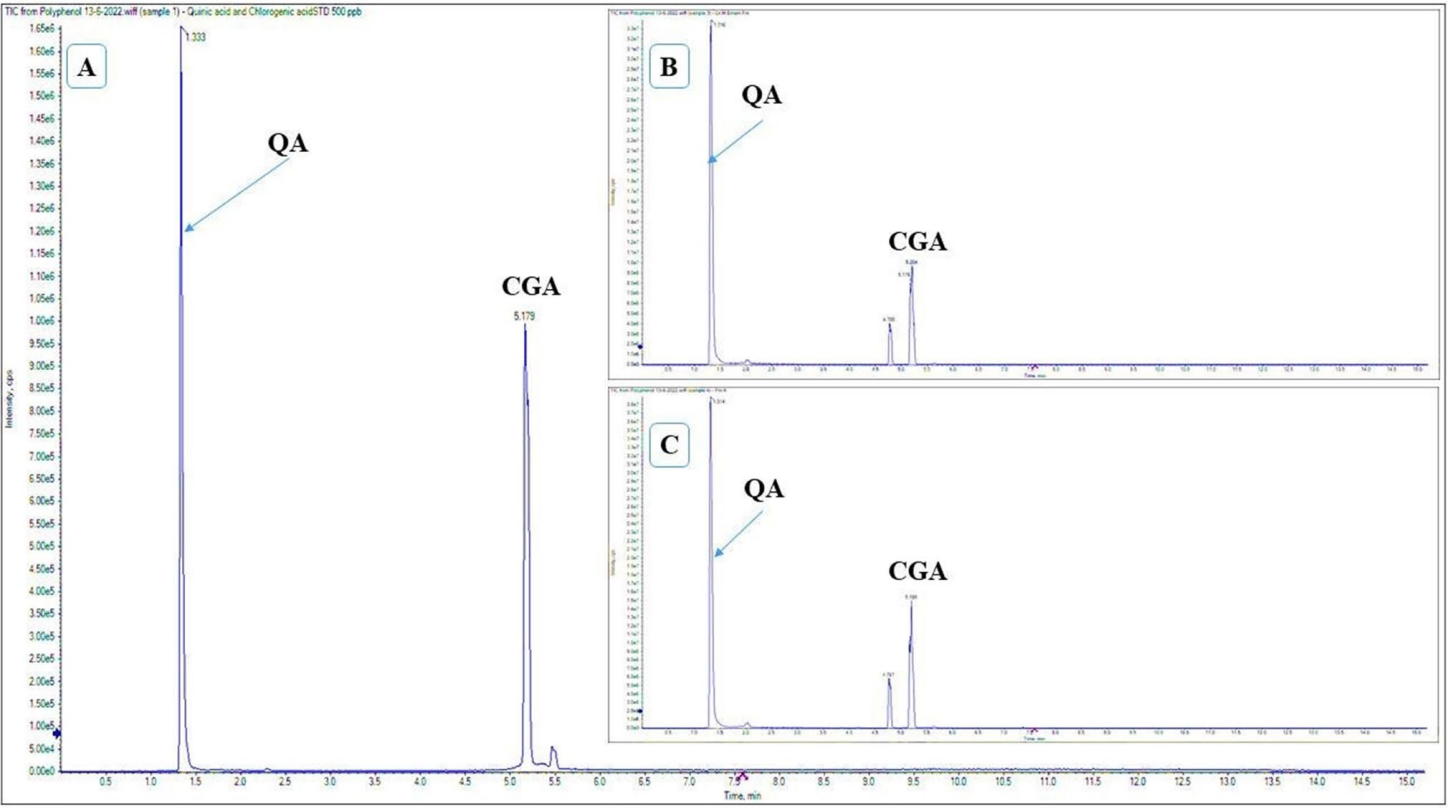
LC–MS chromatograms of (**A)** QA and CGA standard, and (**B)** QA and CGA in *F. drupacea* extract, and (**C)** QA and CGA of FDWF

The content of quinic acid (QA) in the total extract of *F. drupacea* extract and FDWF was found to be 21.12 ± 2.19 and 23.66 ± 2.62 mg/g (Fig. 5), while that of chlorogenic acid (CGA) was 6.30 ± 3.09 and 8.86 ± 0.29 mg/g, respectively.

## Discussion

Ethanol-induced gastric mucosal injury is one of the most frequently used experimental models for evaluating the cytoprotective and antioxidant effects of antiulcer agents (Lahiri and Palit 2012). This model mimics many aspects of acute human gastric mucosal injury condition rats develop sever ulcers, degraded gastric mucosa, increased mucosal permeability, and sometimes bleeding (Aboul Naser et al., 2020). Histopathological and macroscopical examination of the gastric mucosa of ulcerogenic rats revealed severe internal bleeding, as evidenced by severe congestion in the lamina propria submucosa and inter-villus extravasation of RBCs among the gastric mucosal villi (Lustenberger et al. 2011). Also, severe coagulative necrosis was found in some areas of the gastric mucosa (Li et al. 2013). This ulcerogenic condition led to an increase in the levels of inflammatory markers (such as MPO), proinflammatory cytokines (such as TNF-α) and reactive oxygen species (ROS), and a decrease in the levels of the anti-inflammatory cytokines (such as IL-10), PGE2, mucosal enzymes (LDH and SDH), and cellular antioxidants (Brzozowski et al. 2005; Adinortey et al. 2013). AlRashdi et al. 2012 and Kan et al. 2017 reported that increased ROS production and antioxidant depletion are associated with the pathogenesis and progression of ethanol-induced PU, and the accumulated ROS cause lipid peroxidation (Yu et al. 2017).

In this study, ethanol administration increased the level of MDA in the gastric tissues of ulcerogenic rats, but reduced the levels of the antioxidant enzymes SOD and GSH, which is in line with previous findings (Sidahmed et al. 2013).

Pretreatment of ulcerogenic rats with FDWF prevented the ROS-mediated oxidative damage by increasing the activity of the SOD enzyme, restoring the depleted GSH, and decreasing MDA level. It was reported that PGE2 regulates gastric mucus secretion, stimulates blood flow and bicarbonate production, and accelerates healing of ulcers. However, reduced level of PGEs is a relevant marker of mucosal ulceration (Tsuge et al. 2019). The gastroprotective effect of FDWF is mediated, at least in part through increase in the production of gastric IL-10 and PGE2, as supported by the histopathological examination, which revealed reduced inflammatory responses (lower ulcer index). On the other hand, the levels of LDH and SDH were significantly increased after treatment with FDWF or famotidine.

It was reported that the increase in hydrogen ion concentration lowers pH of the gastric juice and promotes gastric damage. FDWF treatment significantly improved gastric pH while decreasing gastric secretion when compared to the ulcerogenic group (Lüllmann et al. 2000). Furthermore, the gastric pH of FDWF-treated or famotidine-treated rats were found to be the same and suggested that FDWF has a strong ability to decrease stomach acid production and neutralize its acidity.

FDWF used in the present study was prepared from the total extract of *F. drupacea* aerial parts. FDWF was rich in phenolic acids (52.3 ± 1.12) and QA and demonstrates promising antioxidant activity (231 ± 0.074 ug/ml). Taking into consideration that ROS are linked to ulcer formation, these constituents could play a role in the anti-ulcer effect of FDWF (Panda and Suresh 2015; Elshamy et al. 2020). Among the phenolic acids identified in FDWF *p*-coumaric acid, caffeic acid, and ferulic acid were reported to increase PGE2 content and mucus formation in the gastric mucosa (De Barros et al. 2008). Also, sinapic acid reduced the severity of ethanol-induced injury of the gastric mucosa through reduction of the gastric acid juice volume and acidity, and increase of PGE2 and NO_2_ levels. These effects were exactly equivalent to those seen for omeprazole. Sinapic acid was also reported to suppress gastric inflammation by lowering MPO, TNF-α, and IL-6, inhibiting lipid peroxidation (MDA), and restoring depleted GSH and CAT activity (Raish et al. 2021). On the other hand, CGA was found to be effective in treating and preventing ethanol/HCl-induced gastric lesions by inhibiting neutrophil migration and restoring the levels of GPx, SOD, CAT, GSH, and TBARS in mice, and prevented the rise of TNF-α and leukotriene B4 (Shimoyama et al. 2013). While QA and its derivatives were reported to exert anti-inflammatory effect by inhibiting the pro-inflammatory markers (Zeng et al. 2009; Sheng et al. 2005).

Accordingly, the gastroprotective and ulcer healing activities of FDWF in this model could be attributed to the antioxidant and anti-inflammatory effects of phenolic acids and QA and its derivatives.

## Conclusion

Gastric mucosal injury is one of the most prevalent gastrointestinal conditions which affect significant segment of the global population. Therefore, developing potent and safe antiulcer medications is needed. For the first time, a phenolic acids-rich fraction (FDWF) was prepared from *F. drupacea* aerial parts and was found to exert a pronounced effect in protecting and treating ethanol-induced gastric mucosal injury in rats. FDWF was standardized and its metabolic profile was clarified using HPLC–ESI–MS/MS. In summary, FDWF is a promising natural product that can be further developed for the management of gastric mucosal injury.

## Data Availability

Data supporting findings are presented within the manuscript. Inquiries about data availability should be directed to the authors.
